# The French System of Medical Education

**Published:** 1864-03

**Authors:** 


### THE FRENCH SYSTEM OF MEDICAL EDUCATION.

In view of the interest excited by the suggestions for the improvement of medical education recently put forth by Professor Syme on behalf of the Scottish branch of the Medical Council, and as it is understood that the subject of further reforms in medical education is likely to be subsequently and warmly discussed by the General Council, it may not be *mal-apropos* that I should give you an outline of the system of study pursued under the direction of the French Government.

There is but one degree for doctors in medicine or in surgery. For this degree five examinations must be undergone before the Faculty. These consist of—1st. Anatomy and physiology, with the test of dissections. 2d. Internal (medical) and external (surgical) pathology, with the performance of operations. 3d. Materia medica, chemistry, and pharmacy. 4th. Hygiene and legal medicine. 5th. Medical and surgical clinical tests, and accouchements. The examinations are public; and the law provides that two of them shall be in Latin, but this has fallen into disuse. After the five examinations, the candidate for a degree must present and defend a thesis written by himself in French or Latin. Before, however, the student can reach the point at which he is admitted to any of these examinations, he has a long ordeal to pass through. Prior to commencing his career by "taking his first inscription" at the Faculty, which is equivalent to what is known as "entering" at the London schools, the student must produce his diploma as *bachelier es lettres*, or bachelor of arts; and before taking his third inscription (the inscriptions are for three months each) he must produce his diploma as bachelor of sciences. A modified diploma of bachelor of sciences is granted to medical students, which is especially adapted for them only. The student usually enters at about the age of twenty or twenty-one. He has to take a fresh inscription every three months, and to pass a special examination at the end of each year for three years. Thus, in addition to the five examinations for the doctorate, there are three annual examinations (*examens de fin d'annee*). These latter bear, first year, on chemistry, physics, and natural history; second year, on anatomy and physiology; third year, on medicine and surgery. Students are not admitted to the fifth, ninth, and thirteenth inscriptions respectfully, unless they have passed with success the "examination of the end of the year." They are only admitted to the examinations for the doctorate after the expiration of the last three monthly periods of the fourth year of study. The official fees for these inscriptions and examinations (independently of those for the diplomas of science and letters are, then, as follows, for the diploma of doctor:—

| | |
|---|---|
| Inscriptions (sixteen, at 30 fr. each) - - | 480 fr. |
| Three examinations at the end of each year (30 fr. each) - - - - | 90 fr. |
| Five examinations, at the conclusion of four years' study, (50 fr. each) - - - | 250 fr. |
| Five certificates of ability (40 fr. each) - | 200 fr. |

| | |
|---|---|
| Thesis, - - - - - - | 100 fr. |
| Certificate of ability, - - - - | 40 fr. |
| Diploma, - - - - - - | 100 fr. |
| | 1260 fr. |

The fees for dissection and for unofficial courses which he has to take come to almost as much.

As to hospital practice, it is prescribed that no one shall be able to take his degree unless he shall have followed the hospital practice either as dresser [externe] or merely as pupil during at least one year. The prescribed curriculum commences after the ninth inscription has been taken—that is to say, at the beginning of the third year; and the four following inscriptions (which are essential in order to be admitted to examination) are not delivered to the student unless he bring with him a certificate of having diligently attended the practice during that time. Thus it will be seen that the French system differs in some material respects from the English. It requires the student to enter a number of courses; but it does not require him to attend them. It only exacts from him that he shall, in a long series of examinations, prove that he has acquired the requisite amount of knowledge. It is essentially a system of examination. The hospital practice need only be registered for one year. As to lectures, they are to be paid for; but they need not be attended, and, practically, they are very irregularly attended. The professors have themselves very much to blame for this, inasmuch as the chairs are habitually employed by their incumbents for the development, at great length, of their views on particular points of the science professed, rather than the systematic and complete teaching of the whole subject in the manner and to the extent required by the student for the purposes of his instruction, and to enable him to pass the stated examinations. The courses of the Faculty, in other words, are by no means squared with the examinations of the Faculty, or with the requirements of the Faculty. Hence the system of education, which is elaborately rounded and perfect to the eye, is, in fact, so imperfect as to require all sorts of supplementary aids. The student does not trust to the professors of the Faculty for his instruction; he trusts to his books and his private teachers. These abound: every one can teach in Paris; and although they are not recognized, these teachers are not the least influential. The Faculty give an unofficial recognition to a number of these private teachers by lending them (for a consideration) the amphitheatres in which to lecture; others are content with lecturing in their private *cliniques*, and some are on the footing of our private grinders. Necessarily with this system of excessive examination, grinding is brought to great perfection; and the whole art of studying the peculiarities of the examiners, and knowing their pet questions and the expected answers, is developed to a greater pitch of completeness in Paris than in London. The last key to the examinations is a little book published by Dr. Berton, (1863), which gives systematic tables of the questions habitually asked at the various examinations, "with the answers of the examiners themselves to the most difficult questions." Some of the general remarks are too amusing to be passed over. "As a general rule," says this astute adviser, "the professors put questions specially on the subjects which they have developed during the year; but the *agreges* examine on all subjects indifferently, unless they have a private *clinique*, or a course at the Ecole Pratique, &c. They also—all of them—manifest a preference for the questions which have especially formed the subjects of their own theses and memoirs. It is useful, then, to know eight days beforehand your series, which can always be ascertained from some one of the secretary's department, or from those of the examiners which may happen to be your hospital teachers. Not only could you thus, under proper circumstances, pay any of them a visit of ceremony, or get yourself privately mentioned to them, but, by a little asking, you will ascertain the kind of questions they put. It will be well also to find out, in point of bibliography, the works which your examiners may have published, and the opinions, more or less personal, they have adopted—an examiner being always more than flattered to find a pupil sufficiently intelligent to reproduce in his presence his favorite opinions, whatever they may be, and the more so if they be little popular amongst the majority of authors. I would advise you, even *strongly*, to follow, during the eight last days —I speak of the third of the annual examinations—the hospital visit of one or other of your examiners, in order to become intimately acquainted with their opinions, and even with their language. They can but be the better disposed to you for doing this. If they have a *clinique* in town, whether free or by payment, enter yourself at this in the same way, even if it be but the evening before the examination.

Of course all this must be taken for what it is worth; but the extreme care with which the list of suggestions are arranged and classified, and the intelligence with which the particular views of the examiners are indicated in notes, sufficiently show that all the weak points of the examiners are diligently studied, and even their special merits used as points of vantage for the pupil. There is one great defect in the French system: it is moulded into an apparent symmetry which is not real. It pretends to be a system of public instruction and public examination. It is, in fact, a system of public examination, but of private instruction. Then the students are obviously over-examined, and must be ground up to a choking point to suit the system. Three annual examinations, five examinations for the doctorate, besides *concours* for the *internats* and the *externats* and the *prix des hopitaux*—travelling over the widest possible range of anatomy, surgery, medicine, physiology, histology, and the accessory sciences—demand an amount of absolute cramming which has a definite an undeniable tendency to produce ultimate mediocrity. I am persuaded that if Mr. Syme were to look into the *Guide et Questionaire* of M. Berton, to which I have referred, his nervous system would sustain a severe shock, and he would immediately desire to "remonstrate with, censure, or, if necessary, depose the whole" body of teachers and examiners of Paris, as loading the memories of the pupils with indigestible details, as well as running through half the rest of his catalogue of pedagogic sins. A complete course of education in Paris is eminently conducive to cerebral congestion, and must load the brains of the pupils with a mass of minute details which no man's memory could retain permanently, and which are only got up for the purpose of the one examination, to be forgotten while preparing for the next. But this is almost an inherent defect in all medical education just now; and, in that it is more particular as to ascertaining that the candidates have the required knowledge than in inquiring where they got it, many persons will think the French system, by so much, more practical than the English, which carefully regulates the means of learning, as well as tests the information acquired.

I must return in a subsequent letter, to the subject of my last—the system of medical education pursued here, and the general arrangements of the hospitals and schools. Meantime, however, not to continue without a break a subject which is not equally interesting to all, I will revert to some of the scientific and practical aspects of medicine as followed here. Of course, medicine must be understood here to include also surgery, for although recognized in fact, the distinctions between surgery and medicine are not accepted in the language or in the theory of the Faculty. Surgeons are all doctors, and physicians have also a qualification in surgery; although M. Trousseau would not think of amputating a leg, and, perhaps, Nelaton would not prescribe for a phthisical patient. Of the latter fact, however, I am not so certain; for since surgeons are also physicians, the etiquette in this respect is by no means rigid, and here, more than in London, would apply the celebrated definition medical and surgical patients ascribed to your "Nestor" of surgery,—Mr. Lawrence,—that "surgical patients were all those patients who paid fees, medical patients all those who did not." The limits between surgery and medicine are obviously altogether artificial and arbitrary; and the difficulty is well got over here by requiring the possession of a joint degree by every practicing physician and surgeon, and leaving it to himself to determine the particular line of practice according to his bent or the force of circumstances. The line is especially difficnlt to draw in obstetric practice, usually followed by physicians in England and elsewhere, but involving a multitude of operative proceedings requiring much surgical skill.

It is precisely of obstetric operative practice that I have to speak in this letter. Dr. Marion Sims, of New York, is now practing in Paris, and as the improvements which he has introduced into what may be called the surgery of females have revolutionized that department of the art all over the world, and his procedures have formed the basis of a new and happy era in the treatment of the series of fistulæ and other diseases of women which were previously incurable, it will be interesting to give some particulars of what he has been doing here surgically since, like many other Americans, he has sought a home in Europe.

Dr. Sims had already achieved a great reputation in America and England by his introduction of silver wire sutures—certainly one of the most important surgical improvements of the century; and by his immediate application of this discovery to the cure of all kinds of fistulæ and clefts in the mucous canals. The success with which he operates on deformities and deficiencies of this kind was not, however, at first fully appreciated in Paris; and when he came over, he was asked to operate on some of the most difficult cases of prolapse of the bladder from sloughing of the tissues, and utero-vesical and vaginal fistulæ, which were known to exist in this capital. He had a triumphant success in these cases, which had been abandoned as hopeless, but which he cured with a single operation; and his reputation soon extended so greatly that he was summoned to the highest and most important personages, and the Emperor has given him a special permission to practice in Paris. It is no breach of confidence, since the fact has already appeared in print, to mention that Dr. Sims has been consulted by the Empress, whose general health is good, but who is known to be delicate.

The great improvements which have constituted Dr. Sims's most precious contributions to obstetric surgery are sufficiently well known in England, but as he is a man who has all his life done much more than he has talked or written about I will only jot down some of the facts which I have gathered as to his practice in some matters of special interest, in which he has, without publishing, anticipated a good deal that has subsequently been published, and forms the theme of discussion.

I pass over other more ordinary fistulæ in which his simple and successful practice, with or without the complications of his pupil Bozeman, is generally followed, to speak of cases of utero-vesico-vaginal fistulæ where the neck of the uterus—more particularly the anterior portion of it—is thoroughly destroyed. Dr. Sims's method of curing the fistula is by bringing its vaginal borders together in such a manner as to shut up the cervix uteri in the cavity of the bladder. The vagina then become a blind pouch. The menses pass into the bladder, mingle with the urine, and the patient passes bloody urine during the menstrual flow. The last operation of this kind which Dr. Sims performed was in Paris, about ten months ago, and in the presence of Dr. Rayer, the late dean of the Faculty, Sir Joseph Oliffe, Dr. M'Carthy, and others. His first operation of this sort was in Alabama, in 1849. I believe it is now a recognized proceeding in London, and Mr. James Lane has lately recorded a singular case in which, even after this operation, the patient became *pregnant*, probably through the medium of some capillary communication left between the vagina and the uterus. I have not seen the details of that case, but in the latter particular it is unique. Dr. Marion Sims has performed the operation more than a dozen times, and has not seen any inconvenience from it.

You published last year a paper by the London surgeon whom I just mentioned—Mr. James Lane, "On Lithotomy in the Female bladder," in favor of the vesico-vaginal incision. Dr. Sims considers that the facility and invariable success with which a cut in the vesico-vaginal septum may now be closed suggest this as "the only justifiable operation for stone in the female bladder." He performed this operation first 1850. It has since been repeatedly performed in America by Dr. Emmett, of the Women's Hospital of New York, and by Dr. Bennett, of Connecticut. The simplicity, safety, and unfailing success of the operation are spoken of in warm terms.

The application of this to the parallel method of *recto-vesical lithotomy in the male*, is a subject worthy of careful consideration. Recto-vesical lithotomy *in the adult* is a proceeding which was used long before the introduction of metallic sutures, and was followed with modifications by Mr. Lloyd, of St. Bartholomew's. Without these sutures it was liable to a serious objection—the occasional persistence of recto-vesical fistula. The silver-wire sutures, however, promise to obviate this inconvenience, Dr. Sims has mentioned to me a case in which Dr. Bauer, of New York, operated by this plan in 1859, Dr. Sims putting in the sutures. He says:—"The patient was placed on the left side, and my speculum was introduced into the rectum, exposing the anterior wall of the rectum, just as it would the vagina in the female. A sound was passed into the bladder. The Doctor entered the blade of a bistoury in the triangular space bounded by the prostrate, the vesiculæ seminales, and the peritoneal reduplication. He passed the finger through this opening, felt the stone, and removed it with the forceps without the least trouble. The operation was done as quickly and as easily as it would have been in a female through the vaginal septum. After the removal of the stone, Dr. Bauer kindly asked me to close the wound with silver sutures, which I did, introducing some five or six wires with the same facility as in the vagina. There was no leakage of urine. The patient recovered without the least trouble of any sort. The wires were removed on the eighth day, and on the ninth day the patient rode in a carriage with Dr. Bauer a distance of four or five miles, to call on and report himself to our distinguished countryman, Dr. Mott. The facility and safety of executing recto-vesical lithotomy (except in children for anatomical reasons), and the success of closing at once the cut by the introduction of metallic sutures, ought to make this the operation in the male."—*London Lancet.*

